# Protocol to study *in vitro* drug metabolism and identify montelukast metabolites from purified enzymes and primary cell cultures by mass spectrometry

**DOI:** 10.1016/j.xpro.2023.102086

**Published:** 2023-02-08

**Authors:** Cátia F. Marques, Pedro F. Pinheiro, Gonçalo C. Justino

**Affiliations:** 1 Centro de Química Estrutural, Institute of Molecular Sciences, Instituto Superior Técnico, Universidade de Lisboa, 1049-001 Lisboa, Portugal

**Keywords:** Metabolism, Mass Spectrometry, Chemistry

## Abstract

We present an optimized protocol set to study the production of drug metabolites in different *in vitro* systems. We detail the necessary steps to identify the metabolites of xenobiotics produced in different metabolic-competent systems, from purified enzymes to primary cell cultures. It is coupled to a high-resolution mass spectrometry analytical approach and can be adapted to study any xenobiotic. This protocol was optimized using montelukast, an antagonist of the cysteinyl leukotriene receptor 1, widely used for asthma management.

For complete details on the use and execution of this protocol, please refer to Marques et al. (2022).^1^

## Highlights

• An *in vitro* set of assays that can be used in drug metabolism studies
• A high-resolution electrospray ionization mass spectrometry analytical tool
• Characterization of drug metabolites by mass spectrometry
• Small-scale metabolite identification

## Abstract (editor highlights)

Publisher’s note: Undertaking any experimental protocol requires adherence to local institutional guidelines for laboratory safety and ethics.

## Graphical abstract

**Figure:**
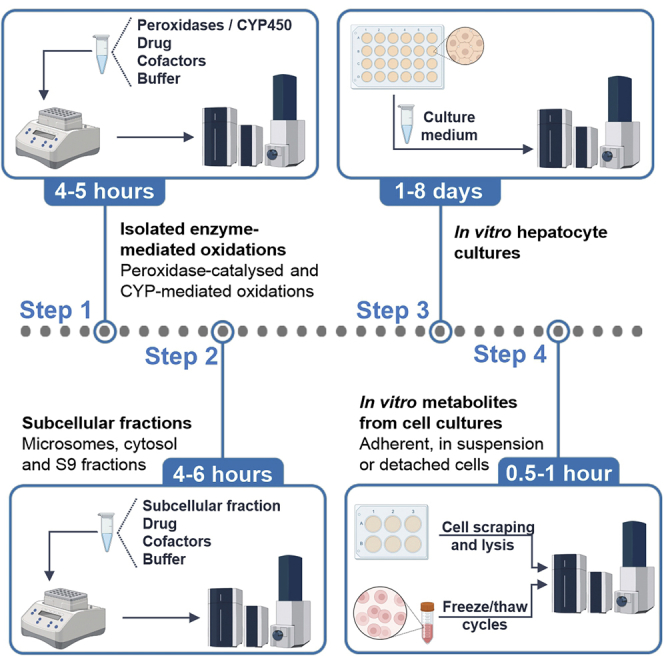

## Before you begin

### Introduction

Xenobiotics are foreign compounds that have no natural physiological activity and that enter the body as food additives, drugs, or pollutants. In order to protect itself against xenobiotics, the body will try to i) prevent their absorption and distribution; ii) promote its physical excretion (e.g., in the urine, in perspiration); or iii) will metabolize them to more water-soluble metabolites that are easier to excrete.^2^^,^^3^ Drug metabolizing enzymes (DMEs) play a crucial role in the metabolism, detoxification, and elimination of drugs. Phase I DMEs are generally responsible for drug oxidation and hydroxylation, while phase II DMES are conjugating enzymes that catalyze the formation of hydrophilic metabolites by conjugating the drugs or drug metabolites with water-soluble groups such as glucuronic or sulfonic acid.^2^^,^^4^^,^^5^ Drug metabolic pathways, generally responsible for drug inactivation, can be used pharmaceutically. For example, pro-drugs are inactive compounds that become active only upon being metabolized in the body.^6^ On the other hand, sometimes drug metabolism leads to the formation of reactive metabolites, that may react with biomolecules, contributing to the onset of toxic adverse drug reactions.^7^^,^^8^ One example is the oxidation of the anti-HIV drug nevirapine to electrophilic metabolites that can form adducts with biomolecules and contribute to nevirapine’s adverse drug reactions.^9^^,^^10^

The elucidation of the metabolic fate of drugs is of paramount importance during the drug development stages, to assess the possible formation of reactive metabolites, and to clarify the biodistribution of the drugs. This protocol describes different assays that can be performed to identify drug metabolites and cover a range of *in vitro* model systems of different complexity in order to explore the metabolite space in the most complete way possible. Assays presented here were tested using montelukast. For complete details on the use and execution of this protocol, please refer to Marques et al.^1^

### Institution permission

All animal procedures were performed in accordance with the EU Directive 2010/63/EU for animal experiments and the Portuguese Law for Animal Care for animal use in research (DL 1/2019), in compliance with the authors’ institutional review board directives (Instituto Superior Técnico, Universidade de Lisboa) and the ARRIVE 2.0 guidelines for planning, conducting, and reporting animal research.^11^

### Enzyme stock solutions

**Timing: 0.5 h**

Commercial enzymes are purchased as powder formulations and stock solutions need to be prepared.

1. Prepare the enzyme stock solutions according to the manufacturer’s indications, to obtain 0.1 U/μL solutions. The mass of each enzyme to weigh depends on the specific activity (U/mg) in each batch.
  a. Horseradish peroxidase (0.1 U/μL): solubilize 8 mg of horseradish peroxidase in 1.7 mL of 0.1 M phosphate buffer pH 7.4. Information available online. This solution can be stored at 4°C for up to 1 week.
  b. Bovine milk lactoperoxidase (0.1 U/μL): solubilize 0.6 mg of lactoperoxidase in 1.2 mL of 0.1 M phosphate buffer pH 7.4. Store at 4°C for up to 1 week.
  c. Mushroom tyrosinase (0.1 U/μL): prepare a stock solution by solubilizing 6.98 mg of tyrosine in 1.0 mL of 0.1 M phosphate buffer pH 7.4. This solution can be stored at −20°C for up to 6 months in small aliquots to prevent multiple thawing. To prepare the working solution, dilute 1 μL of the stock solution in 500 μL of 0.1 M phosphate buffer pH 7.4. Store at 4°C for up to 1 week. Information available online.

***Note:*** sterilization (by filtration) of these solutions is not recommended since it does not increase their shelf-life and can lead to activity loss due to adsorption to filter membranes. Avoid the use of any preservative (e.g., sodium azide) since they can inhibit the enzyme activity.^12^

**CRITICAL:** enzyme solutions, particularly diluted ones should not be stored frozen as this significantly decreases enzyme activity. Assays should be planned in advance to make maximum use of the prepared enzyme solution.

### Preparation of mouse S9 fractions

**Timing: 1–3 h**

**Figure 1:**
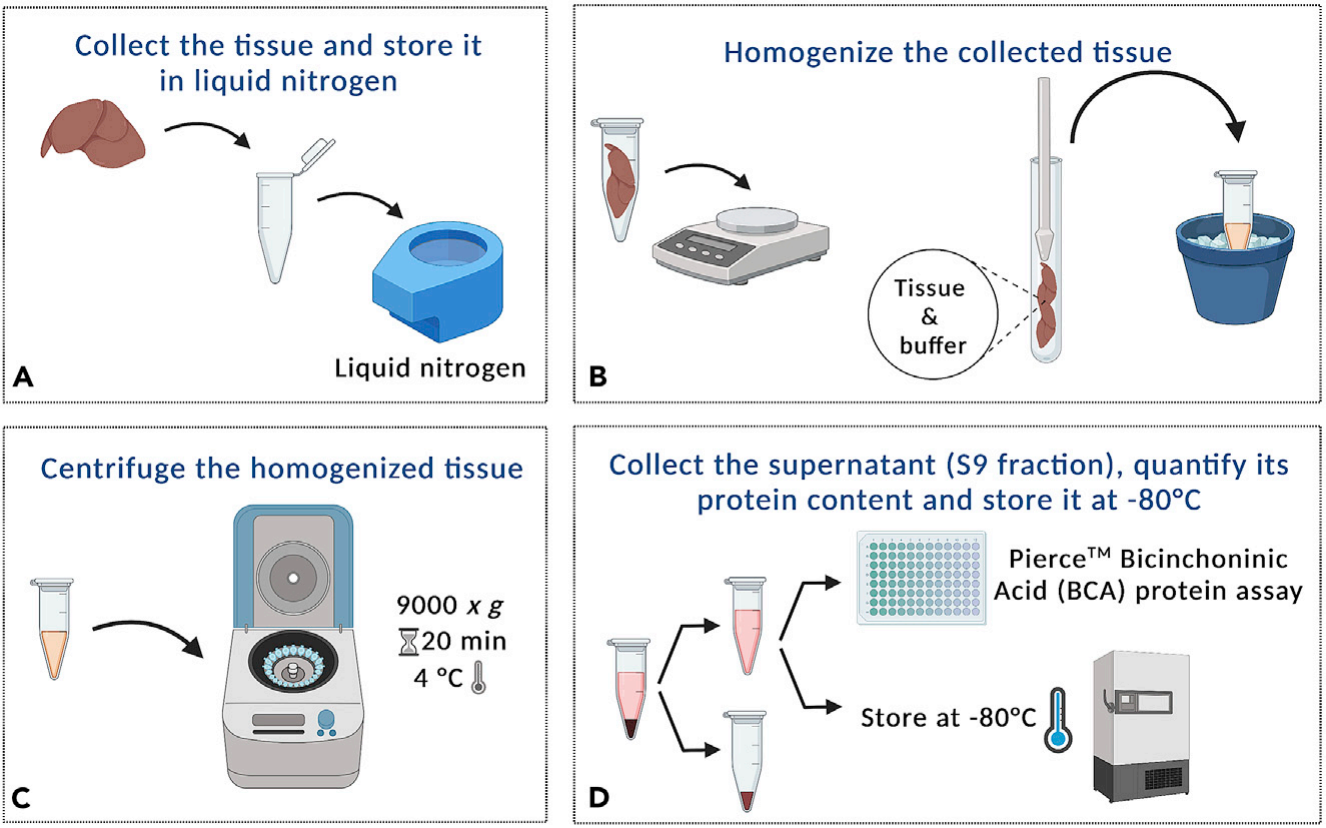
Mouse subcellular fraction isolation protocol (A) Upon anesthesia and euthanasia, selected tissues are collected and stored in liquid nitrogen. (B) Using Potter-Elvehjem tissue homogenizers or micro-pestles, the tissues are homogenized with ice-cold S9 homogenization solution (50 mM Tris-HCl buffer, pH 7.4, supplemented with 150 mM KCl and 2 mM EDTA). (C and D) After centrifugation at 9,000 × *g* for 20 min at 4°C, the supernatant is collected, and their protein content quantified using Pierce™ Bicinchoninic Acid (BCA) protein assay. Supernatants are stored at −80°C until used. Created with BioRender.com.

S9 fractions of tissue homogenates, that contain both cytosol and microsomes, were prepared based on standard methods^13^^,^^14^^,^^15^ (Figure 1). Compared with microsomes or cytosol fractions, the S9 fraction combines the activity of both phase I and II enzymes and can be easily prepared using standard laboratory equipment. Also, small amounts of tissues generally yield large amounts of S9 fraction, and this can be stored at −80°C without significant loss of activity over relatively long periods of time. In terms of representativity, S9 fractions, as well as other subcellular fractions, can be prepared (or acquired) as pools of different donors, covering a larger set of enzyme isoforms, allowing of the identification of more metabolites.

2. Animals must be euthanized according to the national law and institutional guidelines for Animal Care for animal use in research.
  a. Anesthetize the animal with isoflurane and sacrifice by cervical dislocation.
  b. Dissect the tissues of interest (e.g., liver, lungs, kidney, and brain) into microtubes or conical centrifuge tubes.
  c. Store the collected tissues in liquid nitrogen until homogenization.

***Note:*** the anaesthesia and euthanasia methods can be adjusted to the species of interest.

3. Homogenize the collected tissues in ice-cold S9 homogenization solution (50 mM Tris-HCl buffer, pH 7.4, supplemented with 150 mM KCl and 2 mM EDTA) using Potter-Elvehjem tissue homogenizers or micro-pestles. Use 5–10 mL of homogenization solution per gram of frozen tissue.
4. Centrifuge the tissue homogenates at 9,000 × *g* for 20 min at 4°C.
5. Collect the supernatant (S9 fraction) and quantify its protein content using the Pierce™ Bicinchoninic Acid (BCA) protein assay, following the manufacturer’s protocol, available online.
6. Store at −80°C until used.

**CRITICAL:** store the S9 fractions in small volumes to avoid freeze/thaw cycles. The activity of enzymes in solution is quickly lost with 1 or 2 freeze/thaw cycles. Adjust the aliquots volume according to the S9 concentration in order to perform a single use of whole aliquots in one set of experiments, with minimal waste: e.g., a 500 μL aliquot at 20 mg/mL allows preforming 50 assays, while a 200 μL aliquot at 10 mg/mL allows preforming 20 assays.

***Note:*** this protocol was employed to prepare subcellular fractions from mice, but it can be employed to isolate S9 fractions from other species.

### Collagen coating of 24-well tissue culture plates

**Timing: 3–4 h**

7. In a sterile environment (biological safety cabinet) and using sterile 200 μL pipette tips, transfer 200 μL of the collagen coating solution into each well of the 24-well plate.
8. Using a side-to-side sliding motion of the plate, allow the solution to completely cover the bottom of the wells.
9. Cover the plate and transfer it into a 37°C humidified incubator for 2 h.
10. Carefully aspirate the solution from each well, tilting the plate towards the tip.
11. Carefully transfer 0.5 mL of PBS into each well and carefully remove as much as possible and discard.
12. Repeat step 11 one more time.

**CRITICAL:** it is important that all the collagen solution is removed prior to cell seeding as the solvent (acetic acid) will significantly lower the pH of the medium and cause cell death.

13. Plates can be used immediately or allowed to dry in the biological safety cabinet and stored at 4°C for up to one week, tightly covered and sealed with parafilm.

### Hepatocyte seeding preparation

**Timing: 30 min**

14. Decontaminate the laminar flow cabinet (LFC) working surface using a 70% ethanol solution and prepare the working area. All other material (tubes, pipettes, etc.) should be sterilized by autoclave beforehand.
15. Turn on the water bath at 37°C.
16. Warm the hepatocyte culture medium.
17. Fill a 50 mL conical tube with *ca.* 20 mL of warmed hepatocyte culture medium.

## Key resources table

**Table:** 

REAGENT or RESOURCE	SOURCE	IDENTIFIER
**Chemicals, peptides, and recombinant proteins**		

0.9% sodium chloride solution	B. Braun	Cat# 200115
3′-Phosphoadenosine 5′-phosphosulfate (PAPS)	Santa Cruz Biotechnology	Cat# sc-210759
Acetic acid, glacial	Carlo Erba	Cat# 524521
Acetonitrile, Optima™ LC/MS grade	Fisher Scientific	Cat# A955-212
Acetyl coenzyme A (Acetyl-CoA)	Santa Cruz Biotechnology	Cat# sc-210745A
Alamethicin	Sigma	Cat# A5361-500UL
Ammonium bicarbonate, LC-MS grade	Fluka	Cat# 40867-50G-F
Ammonium chloride	Fluka	Cat# 09718
Ammonium hydroxide	Sigma-Aldrich	Cat# 221228
Ammonium hydroxide solution	Honeywell Fluka	Cat# 15645540
Bovine milk lactoperoxidase (E.C. 1.11.1.7)	Sigma-Aldrich	Cat# L2005-10MG
CYP2C8 BACULOSOMES™ Plus Reagent, rHuman	Invitrogen	Cat# PV6138
CYP2D6 BACULOSOMES™ Plus Reagent, rHuman	Invitrogen	Cat# P2283
CYP3A4 BACULOSOMES™ Plus Reagent, rHuman	Invitrogen	Cat# P2377
Dimethylsulfoxide	Sigma-Aldrich	Cat# D8418
EDTA (disodium salt)	Merck	Cat# 8418
Fetal bovine serum, heat inactivated (FBS)	Gibco	Cat# 10500-064
Formic acid, Optima™ LC/MS grade	Fisher Scientific	Cat# A117-50
Gentamicin sulfate solution (10 mg/mL)	BioWittaker	Cat# 17-519Z
Glutathione (GSH)	Panreac AppliChem	Cat# A2084,0005
Glycine	Pronalab	Cat# 940874
Hydrochloric acid, 37%	Sigma-Aldrich	Cat# 258148
HEPES (4-(2-hydroxyethyl)-1-piperazineethanesulfonic acid)	Panreac AppliChem	Cat# A3724
Horseradish peroxidase (E.C. 1.11.1.7)	Sigma-Aldrich	Cat# P8250-5KU https://www.sigmaaldrich.com/deepweb/assets/sigmaaldrich/product/documents/123/532/p8250dat-mk.pdf
Human brain cytosol	Xenotech	Cat# H0001.DC
Human brain S9 fraction	Xenotech	Cat# H0001.DS9
Human insulin solution (10 mg/mL)	Sigma-Aldrich	Cat# I9278
Human pooled liver cytosol	Xenotech	Cat# H0610.C
Human pooled liver microsomes	Gibco	Cat# HMMCPL
Human pooled liver S9 fraction	Gibco	Cat# HMS9PL
Human pooled lung cytosol	Xenotech	Cat# H0610.PC(NS)
Human pooled lung microsomes	Xenotech	Cat# H0610.P(NS)
Human pooled lung S9 fraction	Xenotech	Cat# H0610.PS9(NS)
Hydrocortisone	Sigma-Aldrich	Cat# H0888
Hydrogen peroxide solution 35% (H_2_O_2_)	Sigma	Cat# 1.08600
Liquid nitrogen	Air Liquid	N/A
L-valine ethyl ester hydrochloride (Val-OEt)	Aldrich	Cat# 220698
Magnesium chloride (MgCl_2_)	Aldrich	Cat# 24413-9
Methanol, Optima™ LC/MS grade	Fisher Scientific	Cat# A456-212
Milli-Q water (sterile)	N/A	N/A
Montelukast (MTK)	Carbosynth	Cat# FM137467
Mushroom tyrosinase (E.C. 1.14.18.1)	Sigma-Aldrich	Cat# T3824-50KU https://www.sigmaaldrich.com/deepweb/assets/sigmaaldrich/product/documents/407/287/t3824dat-mk.pdf
Non-essential amino acid mixture solution (100×)	Lonza	Cat# BE13-114E
*N*-α-acetyl-L-cysteine (NAC)	Sigma	Cat# A8199-10G
*N*-α-acetyl-L-lysine (NAL)	Santa Cruz Biotechnology	Cat# sc-284922
Penicillin-streptomycin solution (10,000 units/mL of penicillin and 10,000 μg/mL of streptomycin)	Gibco	Cat# 15140-122
Pierce™ Bicinchoninic Acid (BCA) protein assay	Thermo Scientific	Cat# 23225 https://www.thermofisher.com/document-connect/document-connect.html?url=https://assets.thermofisher.com/TFS-Assets%2FLSG%2Fmanuals%2FMAN0011430_Pierce_BCA_Protein_Asy_UG.pdf
Potassium chloride (KCl)	Sigma-Aldrich	Cat# P3911
Potassium di-hydrogenophosphate (KH_2_PO_4_)	Sigma-Aldrich	Cat# P0662
Rat tail collagen solution (50 μg/mL)	Sigma-Aldrich	Cat# 122-20
Reduced β-nicotinamide adenine dinucleotide phosphate tetrasodium salt (NADPH)	GERBU Biotechnik GMbH	Cat# 1045
*S*-adenosyl-L-methionine (SAM)	CH3 BioSystems	Cat# 890001
Sodium bicarbonate	Fisher Bioreagents	Cat# BP328-500
Sodium chloride (NaCl)	Sigma-Aldrich	Cat# S9888
Sodium hydrogenophosphate (Na_2_HPO_4_)	Sigma-Aldrich	Cat# S9763
Sodium pyruvate solution (100 mM)	Gibco	Cat# 11360-039
TRIS (tris(hydroxymethyl)aminomethane)	Sigma	Cat# 252859-500G
Trypan blue solution (0.4%)	Gibco	Cat# 15250061
Uridine 5′-diphosphoglucuronic acid (UDPGA)	Sigma-Aldrich	Cat# U6751-100MG
Water, Optima™ LC/MS grade	Fisher Scientific	Cat# W6-212
William’s medium E with glutamine, powder	Sigma-Aldrich	Cat# W4125

**Experimental models: Organisms/strains**		

Healthy 10-week-old C57BL/6J mice (5 male and 5 female mice)	Charles Rivers Laboratories	Strain Code 027
Rat (Sprague-Dawley) primary hepatocytes (plateable, male)	ThermoFisher Scientific	Cat# RTCP10 https://www.thermofisher.com/pt/en/home/references/protocols/drug-discovery/adme-tox-protocols/thawing-and-plating-hepatocytes-protocol.html

**Software and algorithms**		

MARS software v3.32	BMG Labtech	N/A
Mzmine v2.51	Katajamaa et al.^16^ Pluskal et al.^17^	https://mzmine.github.io/
ProteoWizard MSConvert	Chambers et al.^18^	https://proteowizard.sourceforge.io/

**Other**		

0.22 μm polyethersulfone (PES) membrane syringe filter	Branchia	Cat# SFPE-22E-050
Autosampler glass vials with caps and inserts	N/A	N/A
Centrifuge conical tubes, 15 mL (sterile)	Orange scientific	Cat# 4440310
Centrifuge with swinging-bucket rotor	MPW Med. Instruments	Cat# MPW-380
CO_2_ incubator	Heal Force	Cat# HF90
Eppendorf ThermoMixer® C	Eppendorf	Cat# 5382000015
Hemocytometer	Hirschmann	Cat# 8100204
High-resolution mass spectrometer	Bruker	Impact II
HPLC column, Luna 3 μm C18(2) (100 Å, 150 × 2 mm)	Phenomenex	Cat# 00F-4251-B0
Ice box (Styrofoam)	N/A	N/A
Inverted phase-contrast microscope	Optika	IM-3
Micro-pestles	Carl Roth	Cat# CXH7.1
Microtube centrifuge, refrigerated	Gyrozen	Cat# 1730R
Microtubes (Safe-Lock, polypropylene), 1.5 mL and 2 mL	Eppendorf	Cat# 0030120086 Cat# 0030120094
Parafilm® M	Marienfeld	Cat# 7403810
Pipette controller	Bellco Glass	Cat# BELC1225-80122
Pipettes, variable volume, 1–10 μL, 20–200 μL, 100–1000 μL	Eppendorf	Research Plus
Potter-Elvehjem homogenizer, 3 mL	Sigma	Cat# P7734
Refrigerated microtube centrifuge	Gyrozen	1730R
SecurityGuard Cartridges, C18 4 × 2.0 mm ID	Phenomenex	Cat# AJ0-4286
Serological pipettes, 10 mL	Sarstedt	Cat# 86.1688.010
Serological pipettes, 25 mL	Sarstedt	Cat# 86.1685.020
Serological pipettes, 5 mL	Sarstedt	Cat# 86.1687.010
SPECTROstar BMG Labtech equipped with a multiwell plate reader and a low-volume microspot plate	BMG Labtech	SpectrostarNano
Swing-bucket centrifuge	VWR	Mega Star 600R
Syringes, 5 mL	B.Braun	Cat# 4606051V
Tissue culture plates, 24 wells	Sarstedt	Cat# 83.3922
UHPLC system	Bruker	Elute LC
Water bath, thermostatized	LBX Instruments	N/A

**CRITICAL:** many chemicals used in this protocol, such as acetonitrile, acetic acid, ammonium hydroxide, formic acid, hydrochloric acid, and methanol, are highly flammable, potentially harmful and irritant to eye, skin, and respiratory tract. Handle these reagents in a fume hood, and use appropriate personal protection equipment including lab coat, gloves and safety glasses.

## Materials and equipment

All culture media and solutions prepared were found to be stable for at least 6 months, unless stated otherwise. Deterioration of liquid media and other solutions may be recognized, among others, by color change, formation of precipitates or particulates, or a cloudy appearance.

### Primary hepatocyte cultures

**Table:** William’s medium E with glutamine

Reagent	Final concentration	Amount
William’s medium E with glutamine	N/A	10.8 g
Sodium bicarbonate	2.2 g/L	2.2 g
Milli-Q water	N/A	Up to 1 L
**Total**	**N/A**	**1 L**

***Note:*** Sterilize by filtration and store at 4°C; stable for at least 6 months.

**Table:** Hepatocyte culture medium

Reagent	Final concentration	Amount
William’s medium E with glutamine	N/A	424.5 mL
FBS	10% (v/v)	50 mL
Non-essential amino acids solution (100 ×)	1% (v/v)	5 mL
Sodium pyruvate solution (100 mM)	1 mM	5 mL
Penicillin-streptomycin solution (10,000 units/mL / 10,000 μg/mL)	100 units/mL / 100 μg/mL	5 mL
Amphotericin solution (250 μg/mL)	100 μg/mL	0.5 mL
Gentamicin sulfate solution (10 mg/mL)	40 μg/mL	2 mL
Hydrocortisone solution (1.4 mM)	1.4 μM	0.5 mL
HEPES solution (1 M)	15 mM	7.5 mL
Human insulin solution (10 mg/mL)	32 U/mL	56 μL
**Total**	**N/A**	**≈ 500 mL**

***Note:*** Prepared from sterile solutions under aseptic conditions; stable for at least 6 months at 4°C. Complete medium should look pale orange and should be discarded if the colour changes or precipitates are formed.

**Table:** Phosphate buffer saline (PBS)

Reagent	Final concentration	Amount
Sodium chloride (NaCl)	137 mM	8 g
Potassium chloride (KCl)	2.7 mM	0.2 g
Sodium hydrogen phosphate (NaHPO_4_)	10 mM	1.44 g
Potassium di-hydrogen phosphate (KH_2_PO_4_)	1.8 mM	0.245 g
Milli-Q water	N/A	1 L
**Total**	**N/A**	**1 L**

***Note:*** Dissolve the solids in approx. 800 mL of water, adjust the pH to 7.4 with 1 M NaOH or 1 M HCl, complete volume to 1 L and sterilize by autoclave (15 min, 121°C). Store at 22°C–24°C. PBS solutions can be used indefinitely as long as they do not show signs of microbial contamination.

**Table:** Other solutions

Name	Reagents
HEPES solution	1 M HEPES in milli-Q water. Sterilize by filtration and store at −20°C in 10 mL aliquots for up to 2 years.
Hydrocortisone solution	1.4 mM hydrocortisone in ethanol. Add 5 mg of hydrocortisone in 10 mL of ethanol. Sterilize by filtration (0.22 μm) and store at −20°C in 0.5 mL aliquots. Stable for at least 2 years. Discard if thawed.
Trypan blue solution 0.1%	0.1% trypan blue solution in PBS. Add 1 mL of 0.4% trypan blue solution to 3 mL of PBS. Filter through a 0.22 μm syringe filter to remove suspension solids. Store at 22°C–24°C protected from light. Can be used as long as no contamination is observed.

### Mouse subcellular S9 fraction isolation protocol

**Table:** S9 homogenization solution: 50 mM Tris-HCl buffer, pH 7.4, supplemented with 150 mM KCl and 2 mM EDTA

Reagent	Final concentration	Amount
TRIS	50 mM	1.21 g
Potassium chloride (KCl)	150 mM	2.24 g
EDTA	2 mM	0.15 g
Milli-Q water	N/A	Up to 200 mL
**Total**	**N/A**	**200 mL**

***Note:*** Adjust pH to 7.4 before the addition of potassium chloride and EDTA; store at 4°C; stable for at least 6 months or until contaminated. Buffers at this molarity range are prone to fungal contamination, easily recognizable by the appearance of fluffy particles in solution.

### Metabolism assays

The different buffers and cofactors solutions used in the preparation of reaction mixtures are described below. Most solutions are prepared in ammonium bicarbonate buffers to minimize the presence of MS-incompatible salts in the final samples.

**Table:** 

Name	Reagents
50 mM ammonium bicarbonate (NH_4_HCO_3_) buffer, pH 7.4	50 mM ammonium bicarbonate (NH_4_HCO_3_, LC/MS grade) in milli-Q water. Add 1.98 g of ammonium bicarbonate in 500 mL of milli-Q water. Adjust the pH to 7.4 with hydrochloric acid or ammonium hydroxide (NH_4_OH), as necessary. Store at 22°C–24°C for up to 2 months.
50 mM ammonium bicarbonate (NH_4_HCO_3_) buffer, pH 6.7	Use 5 mL of 50 mM ammonium bicarbonate (NH_4_HCO_3_, LC/MS grade) buffer, pH 7.4, and adjust the pH to 6.7 with hydrochloric acid or ammonium hydroxide (NH_4_OH), as necessary. Store at 22°C–24°C for up to 2 months.
300 mM ammonium bicarbonate (NH_4_HCO_3_), pH 7.4	300 mM ammonium bicarbonate (NH_4_HCO_3_, LC/MS grade) in milli-Q water. Add 11.86 g of ammonium bicarbonate in 500 mL of milli-Q water. Adjust the pH to 7.4 with hydrochloric acid or ammonium hydroxide (NH_4_OH), as necessary. Store at 22°C–24°C for up to 2 months.
1 M hydrogen peroxide	Dilute a commercial hydrogen peroxide 35% solution in milli-Q water. Add 0.565 mL of hydrogen peroxide to 4.435 mL of water. Prepare fresh before use.
3 M potassium chloride	3 M KCl in milli-Q water. Add 0.89 g of potassium chloride in 4 mL of milli-Q water. Store at 22°C–24°C for at least 6 months.
20 mM NADPH	20 mM NADPH in 50 mM NH_4_HCO_3_ buffer, pH 7.4. Add 7 mg of NADPH in 411 μL of 50 mM NH_4_HCO_3_ buffer, pH 7.4. Store at −20°C. This solution can be used until it shows no color change. Deterioration of the solution may be recognized by color changes to yellow, first to very pale hues and later to a stronger yellow color.
250 mM UDPGA	250 mM UDPGA in 50 mM ammonium bicarbonate buffer, pH 7.4. Add 32 mg of UDPGA in 200 μL of 50 mM ammonium bicarbonate buffer, pH 7.4. Store at −20°C up to 6 months.
5 mM PAPS	5 mM PAPS in 50 mM ammonium bicarbonate buffer, pH 7.4. Add 0.5 mg of PAPS in 200 μL of 50 mM ammonium bicarbonate buffer, pH 7.4. Store at −20°C up to 6 months.
400 mM MgCl_2_	400 mM MgCl_2_ in 50 mM ammonium bicarbonate buffer, pH 7.4. Add 7.7 mg of MgCl_2_ in 200 μL of 50 mM ammonium bicarbonate buffer, pH 7.4. Store at −20°C up to 6 months.
50 mM GSH	50 mM glutathione in 50 mM ammonium bicarbonate buffer, pH 6.7. Add 3 mg of GSH in 200 μL of 50 mM ammonium bicarbonate buffer, pH 6.7. Store at −20°C. Solution can be used until it shows visible signs of deterioration. Deterioration of the solution may be recognized by color changes to yellow.
200 mM NAC	200 mM *N*-α-acetyl-L-cysteine in 50 mM ammonium bicarbonate buffer, pH 7.4. Add 6.5 mg of NAC in 200 μL of 50 mM ammonium bicarbonate buffer, pH 7.4. Store at −20°C up to 6 months.
200 mM NAL	200 mM *N*-α-acetyl-L-lysine in 50 mM ammonium bicarbonate buffer, pH 7.4. Add 7.5 mg of NAL in 200 μL of 50 mM ammonium bicarbonate buffer, pH 7.4. Store at −20°C up to 6 months.
200 mM Val-OEt	200 mM L-valine ethyl ester hydrochloride in 50 mM ammonium bicarbonate buffer, pH 7.4. Add 7.3 mg of Val-OEt in 200 μL of 50 mM ammonium bicarbonate buffer, pH 7.4. Store at −20°C up to 6 months.
50 mM Acetyl-CoA	50 mM acetyl-coenzyme A in 50 mM ammonium bicarbonate buffer, pH 7.4. Add 5 mg of Acetyl-CoA in 112 μL of 50 mM ammonium bicarbonate buffer, pH 7.4. Store at −20°C up to 2 weeks.
1 mM glycine	200 mM glycine in 50 mM ammonium bicarbonate buffer, pH 7.4. Add 3 mg of glycine in 200 μL of 50 mM ammonium bicarbonate buffer, pH 7.4. Store at −20°C up to 6 months.

**Table:** *In vitro* metabolites from cell culture

Name	Reagents
Methanol:Water solution (4:1, v/v)	Prepare a solution with 20 mL of methanol (LC/MS grade) and 5 mL of water (LC/MS grade). Store at 4°C up to 6 months.
Methanol:acetonitrile:water solution (2:2:1, v/v/v)	Prepare a solution with 10 mL of methanol (LC/MS grade), 10 mL of acetonitrile (LC/MS grade) and 5 mL of water (LC/MS grade). Store at 4°C up to 6 months.

## Step-by-step method details

In addition to the assays described, the corresponding controls should also be prepared, as indicated for each enzymatic system. Unless stated otherwise, enzymes and enzyme-containing preparations are kept on ice, and all preparations are performed at 22°C–24°C.

### Isolated enzyme-mediated oxidations

**Timing: 4**–**6 h (variable depending on the number of assays prepared)**

This describes the detailed protocol for the incubation of drugs with isolated enzymes in order to identify drug metabolites. A range of peroxidases are used, as these offered simple model oxidation systems to simulate phase I oxidation reactions. Recombinant human cytochrome P450 are used as baculosomes. Baculosomes are a brand of microsomes containing insect expressed recombinant human CYP isoforms co-expressed with recombinant human NADPH:CYP oxidoreductase. Three CYP isoforms are tested: CYP2C8, CYP2D6, and CP3A4. Other isoforms may be available, and the choice depends on the expected relevance of each one. These three isoforms account for a large majority of CYP-mediated reactions.

#### Peroxidase-catalyzed oxidations

**Timing: 4**–**5 h**

This procedure provides detailed steps in order to prepare reaction mixtures that allow the production of phase I drug metabolites by a range of peroxidase.

1. Prepare the peroxidase stock solutions according to the manufacturer’s instructions (see enzyme stock solutions).

***Note:*** acquired enzymes must be stored adequately following the product information (typically at −20°C, or as indicated by the manufacturer) in order to minimize compromising enzymatic activity.

**CRITICAL:** enzyme working solutions must be stored at 4°C and not be frozen. Activity decays with time.

2. On a labeled microtube, add the adjusted volume of 50 mM ammonium bicarbonate buffer pH 7.4 to prepare a 200 μL reaction.
3. Add 25 μM of your drug to each microtube.
4. Add the required cofactors:
  a. 100 mM hydrogen peroxide to horseradish peroxidase or bovine milk lactoperoxidase.
5. Complete the reaction mixture with 1 μL (0.1 U) of enzyme solution and mix components by pipetting the reaction mixture up and down.

The table below summarizes the components present in the reaction mixtures. See table **Reaction mixtures of peroxidase-catalyzed reactions**.

**CRITICAL:** pH variation is one of the key factors that can affect enzyme activity. All reactions and incubations in this protocol are performed with reaction mixtures in which the buffer solution accounts for at least 7/8 of the total volume.

***Note:*** enzyme activity is also frequently affected by the environmental (solvent) composition. It is a good practice to maintain the proportion of different components approximately constant. For example, for drugs that must be dissolved in organic solvents, such as DMSO, if performing reactions with different drug concentrations, initial drug solutions with different concentrations should be prepared, so that the final DMSO/water ratio is maintained.

**Table:** Reaction mixtures of peroxidase-catalyzed reactions

	Horseradish peroxidase	Bovine milk lactoperoxidase	Mushroom tyrosinase	Final concentration
50 mM ammonium bicarbonate buffer, pH 7.4	178 μL	178 μL	198 μL	N/A
Enzyme stock solution (0.1 U/μL)	1 μL	1 μL	1 μL	0.1 U
5 mM drug solution	1 μL	1 μL	1 μL	25 μM
1 M H_2_O_2_	20 μL	20 μL	–	100 mM
**Total volume**	**200 μL**	**200 μL**	**200 μL**	**N/A**

6. Prepare controls for each peroxidase – drug pair by replacing the enzyme with the same volume of reaction buffer (50 mM ammonium bicarbonate buffer). These controls will allow assessing the formation of possible non-enzymatic drug derivatives in each system.
7. Incubate the reaction mixtures for 3 h at 37°C under very mild agitation. See troubleshooting 1.

***Note:*** the incubation time was optimized for MTK. However, other incubation periods can be used.

8. Collect 100 μL from the reaction mixture and analyze by UPLC-MS/MS. See troubleshooting 2.

**CRITICAL:** if the drug has a low protein binding profile, the reactional mixture can be quenched with 50 μL of cold acetonitrile, vortex for 10 s, and centrifuged at 16,000 × *g* for 10 min at 4°C to pellet the proteins out of solution. If your drug is known or suspected to bind strongly to proteins, do not quench the reaction.

#### Cytochrome P450-mediated oxidations

**Timing: 4–6 h**

Phase I metabolites can also be assessed using recombinant human cytochrome P450. A detailed set of steps is described below.

9. Thaw baculosomes according to the manufacturer’s instructions.
  a. Baculosomes should be thawed rapidly in a 37°C water bath and kept on ice until use.

***Note:*** store (−80°C for up to 6 months) and thaw the subcellular fractions adequately in order to minimize compromising enzymatic activity. On first thawing, prepare 50 μL aliquots of the baculosomes’ suspensions.

10. On a labeled microtube, add the required volume of 50 mM ammonium bicarbonate buffer pH 7.4 to prepare a 100 μL reaction (see Table below).
11. Add the drug to each microtube to a final 25 μM concentration.
12. Add the adequate cofactors: 5 μL of 20 mM NADPH, for a final 1 mM concentration.

***Note:*** we add a large excess of reduced NADPH to guarantee the availability of the reduced cofactor throughout the whole assay. Alternatively, an *in situ* NADPH production system can be employed, making use of the glucose-6-phosphate oxidation to the corresponding lactone by glucose-6-phosphate dehydrogenase with the concomitant reduction of NADP^+^ to NADPH. However, commercial kits for this are usually phosphate buffered, and thus non-MS compatible. In alternative, MS-compatible formulations of the regeneration system can be developed.

13. On a labeled microtube, add 10 pmol of CYP4A4 or CYP2C8, or 20 pmol of CYP2D6. For a 1,000 pmol/mL CYP baculosome preparation, pipette 10 μL of CYP4A4 or CYP2C8 baculosomes, or 20 μL of CYP2D6 baculosomes.

***Note:*** baculosomes are supplied with information regarding their protein content, that varies between lots. The baculosomes we used typically have a total 1,000 pmol/mL total CYP content, with 200–300 pmol of CYP per mg of total protein.

***Note:*** a commercial alternative to the protocol followed in this work is available, the Vivid® CYP450 Screening Kits. This kit allows assessing the metabolism and the inhibition of human CYP450 isozymes through a high-throughput screening in multiwell plates, with or without the fluorescent component of the assay. Instructions are available online (https://www.thermofisher.com/document-connect/document-connect.html?url=https://assets.thermofisher.com/TFS-Assets%2FLSG%2Fbrochures%2FVividScreeningKitManual24Apr20121.pdf). However, this kit uses phosphate buffers, whereby it is not suitable for mass spectrometry analysis.

***Note:*** powder and lyophilized formulations of CYPs are also available. However, we have observed that the baculosome formulation offers higher reproducibility between assays and allows assays with longer incubations when required.

The table below summarizes the components present in the reaction mixtures. See table **Reaction mixtures of CYP450-mediated oxidations**.

**Table:** Reaction mixtures of CYP450-mediated oxidations

	CYP2C8	CYP2D6	CYP3A4	Final concentration
50 mM NH_4_HCO_3_ buffer, pH 7.4	84.5 μL	74.5 μL	84.5 μL	N/A
Baculosomes stock (CYP450 content: 1,000 pmol/mL)	10 μL	20 μL	10 μL	10 pmol (CYP2C8 and CYP3A4) or 20 pmol (CYP2D6)
5 mM drug	0.5 μL	0.5 μL	0.5 μL	25 μM
20 mM NADPH	5 μL	5 μL	5 μL	1 mM
**Total volume**	**100 μL**	**100 μL**	**100 μL**	**N/A**

14. Prepare controls for each baculosome – drug pair by replacing the enzyme with the same volume of reaction buffer (50 mM ammonium bicarbonate buffer). These controls will allow assessing the formation of possible non-enzymatic drug derivatives in each system.
15. Incubate the reaction mixtures for at 37°C for 3 h. See troubleshooting 1.

***Note:*** the incubation time was optimized for MTK. However, other incubation periods can be performed.

16. Collect 100 μL from the reaction mixture and analyze by UPLC-MS/MS. See troubleshooting 2.

**CRITICAL:** if the drug has a low protein binding, the reactional mixture can be quenched with 50 μL of cold acetonitrile, vortex for 5 s, and centrifuged at 16,000 × *g* for 10 min at 4°C to pellet the proteins out of solution. If your drug is known or suspected to bind strongly to proteins, do not quench the reaction.

### Metabolic assays using subcellular fractions

**Timing: 4–6 h**

***Note:*** The subcellular fractions used in metabolism assays should be chosen taking into account the organism that we are interested in. Different subcellular fractions are available commercially, including fractions from human, mouse, rat, and rabbit, among other.

Subcellular fractions allow the differential study of phase I and phase II metabolism. According to the cofactors used, it is possible to combine different reactions (e.g., oxidation and glucuronidation) in just one reactional mixture. A detailed protocol is provided below.

17. Thaw the subcellular fractions according to the manufacturer. Microsomes, as well as cytosol and S9 fractions, should be thawed slowly on ice and kept on ice until use.

***Note:*** store (−80°C for up to 6 months) and thaw the subcellular fractions adequately in order to minimize compromising enzymatic activity. On first thawing, prepare 50 μL aliquots of the subcellular fractions’ suspensions.

18. On a labeled microtube, add the required volume of 50 mM ammonium bicarbonate buffer pH 7.4 to prepare a 100 μL reaction.

**CRITICAL:** some subcellular fractions can have lower protein concentration. When the volume of S9 fraction is higher than 10% of the reaction volume, reactions are performed with 50 μL of 300 mM buffer and the total 100 μL volume was fulfilled with water.

19. Add the drug to each microtube, to a final 25 μM concentration.
20. Add the adequate cofactors, as indicated in the tables presented below: **Metabolic reactions to determine phase I and II metabolism** and **Example of some reaction mixtures using phase I and phase II metabolism reactions**.
21. Add microsomes, cytosol, or S9 fraction for a total protein concentration of 1 mg of protein per mL of reaction mixture.

***Note:*** the concentration of a specific enzyme in each fraction varies within lots. By using this approach, we are not focusing on the quantitative aspects of the assay but only the qualitative side of drug metabolite identification.

**Table:** Metabolic reactions to determine phase I and II metabolism

	Reactions	Subcellular fractions	Cofactors
Phase I	Oxidation	CYP isoforms	1 mM NADPH
		S9 fraction	
		Cytosol	
		Microsomes	
Phase II	Glucuronidation	S9 fraction	5 mM UDPGA and 25 μg/mL alamethicin∗^19^
		Microsomes	
	Sulfonylation	S9 fraction	50 μM PAPS
		Cytosol	
	Methylation	S9 fraction	200 μM SAM and 2 mM MgCl_2_
		Cytosol	
		Microsomes	
	Conjugation with GSH or other sulfur compounds	S9 fraction	2.5 mM GSH 1 mM NAC
		Cytosol	
		Microsomes	
	Aminoacid conjugation	S9 fraction	1 mM NAL 1 mM Val-OEt 2.5 mM Acetyl-CoA and 1 mM glycine
		Cytosol	
		Microsomes	

CYP, cytochrome P450; GSH, glutathione; MgCl_2_, magnesium chloride; NAC, N-α-acetyl-L-cysteine; NADPH, reduced β-nicotinamide adenine dinucleotide phosphate; NAL, N-α-acetyl-L-lysine; PAPS, 3′-phosphoadenosine 5′-phosphosulfate; SAM, S-adenosyl-L-methionine; UDPGA, uridine 5′-diphosphoglucuronic acid; Val-OEt, L-valine ethyl ester hydrochloride.

∗Alamethicin is a peptide used as membrane disrupting agent to increase the permeability of endoplasmic reticulum membranes where the UDP-glucuronosyltransferases are located.^19^

The table below exemplifies some reaction mixtures used for the study of the metabolic fate of montelukast. Some of the metabolites produced using these systems are presented in expected outcomes.

**Table:** Example of some reaction mixtures using phase I and phase II metabolism reactions

	Oxidation & glucuronidation	Methylation	Oxidation & conjugation with GSH	Conjugation with GSH	Glucuronidation	Final concentration
	Human liver microsomes: 20 mg/mL	Human liver cytosol: 10 mg/mL	Human liver S9 fraction: 20 mg/mL	Human brain cytosol: 4 mg/mL	(*In house*) mouse liver S9 fraction: 10.15 mg/mL	
300 mM NH_4_HCO_3_ buffer, pH 7.4	–	50 μL	–	50 μL	50 μL	N/A
50 mM NH_4_HCO_3_ buffer, pH 7.4	87.5 μL	–	84.5 μL	–	–	N/A
Milli-Q water	–	29 μL	–	20 μL	37.5	N/A
Subcellular fraction	5 μL	10 μL	5 μL	25 μL	10 μL	1 mg/mL
5 mM drug	0.5 μL	0.5 μL	0.5 μL	0.5 μL	0.5 μL	25 μM
20 mM NADPH	5 μL	–	5 μL	–	–	1 mM
250 mM UDPGA	2 μL	–	–	–	2 μL	5 mM
2 mM SAM	–	10 μL	–	–	–	200 μM
400 mM MgCl_2_	–	0.5 μL	–	–	–	2 mM
50 mM GSH	–	–	5 μL	5 μL	–	2.5 mM
Total volume	100 μL	100 μL	100 μL	100 μL	100 μL	N/A

22. Prepare controls for each subcellular fraction – drug pair by replacing the enzyme with the same volume of reaction buffer (50 mM ammonium bicarbonate buffer). These controls will allow assessing the formation of possible non-enzymatic drug derivatives in each system.
23. Incubate the reaction mixtures for 3 h at 37°C with very mild agitation. See troubleshooting 1.

***Note:*** the incubation time was optimized for MTK. However, other incubation periods can be used.

24. Collect 100 μL from the reaction mixture and analyze by UPLC-MS/MS. See troubleshooting 2.

**CRITICAL:** if the drug has a low protein binding, the reactional mixture can be quenched with 50 μL of cold acetonitrile, vortex for 5 s, and centrifuged at 16,000 × *g* for 10 min at 4°C to pellet the proteins out of solution. If your drug is known or suspected to bind strongly to proteins, do not quench the reaction.

### *In vitro* hepatocyte cultures

**Timing: 24 h–8 days**

As the liver is the major site of drug metabolism, hepatocytes cultures are one of the *in vitro* approaches that can mimic *in vivo* liver metabolism. Primary hepatocytes can be seeded onto 2D supports or 3D reactors and incubated with the drugs under study. Although 3D cultures can better mimic the whole cell activity of the organ, allowing for the observation of more complex interactions between xenobiotics and the biotransformation machinery, 2D systems are cheaper and simpler to use. Conventional adherent hepatocyte cultures require lower number of cells and do not demand highly specialized equipment, while sufficing for metabolite identification.^10^ However, it is important to consider that these cultures are short-lived (when compared to 3D systems) and, therefore, assays must be planned considering this. One approach that extends the culture life span is the use of collagen-coated culture surfaces. This allows for a better attachment of the cells and provide conditions closer to *in vivo* environment, being helpful for maintaining specific hepatic functions.^20^

#### Hepatocyte seeding and treatment

**Timing: 24 h–8 days**

Primary hepatocytes can be freshly isolated by liver perfusion or acquired from various suppliers. A detailed isolation protocol can be found in the literature.^21^^,^^22^ In this work, we used commercial primary hepatocytes, described in detail in the next steps.

***Note:*** not all the commercial primary hepatocytes are suitable for plating. Before planning an experiment, confirm you are buying the most suitable lot according to your experimental plan.

Primary hepatocyte samples should be thawed according to indicated by the manufacturer. In the case of the RTCP10 hepatocytes used in this work, instructions are available online. These hepatocytes can be used immediately or maintained in culture in order to determine the metabolic activity during a period of time. Here, we describe the procedure for cell maintenance and use for drug metabolism studies. See hepatocyte seeding preparation.

25. Quickly thaw the vial containing cryopreserved hepatocytes in a 37°C water bath.
26. Decontaminate the outside of the vial using 70% ethanol.
27. Using a 1 mL micropipette, transfer the content of the vial into a centrifuge tube containing *ca.* 20 mL of warmed hepatocyte culture medium.
28. Applying slow inversion movements, disperse the cells into the hepatocyte culture medium.
29. Pellet the cells by centrifugation at 50 × *g* for 5 min, at 22°C–24°C.
30. Discard the supernatant and, slowly and carefully, resuspend the cell pellet in 3–5 mL of hepatocyte culture medium.

***Note:*** as some samples of primary hepatocytes may contain clumped cells, it is important to use wide-bore tips. If unavailable, a box of 1,000 μL tips with cut ends may be prepared in advance and sterilized in the autoclave.

31. Collect a sample (approx. 50 μL of cell suspension) and determine cell number and viability using a hemocytometer and the trypan blue exclusion method. See troubleshooting 3.
32. Adjust the density of the cell suspension to 600,000 viable cells/mL.
33. Transfer 500 μL of cell suspension to each well of the collagen-coated plates (300,000 cells per well). Cover the plate and transfer it into the incubator (37°C, 5% CO_2_ in humidified air). See collagen coating of 24-well tissue culture plates.
34. Holding the plate flat against the incubator shelf with your dominant hand, move the plate three times left to right and three times forwards and backwards, to evenly distribute the cell suspension over the coated surface.

**CRITICAL:** these movements must be done carefully to avoid damaging the cells.

35. Leave to incubate undisturbed for 24 h for cells to adhere. See troubleshooting 4.
36. Following the initial 24 h-incubation, cultures are examined under phase-contrast. See troubleshooting 5.

***Note:*** healthy cultures should appear as a homogeneous layer of round cells firmly attached to the bottom of the plate.

37. Prepare an incubation of the seeded hepatocytes with the drug of interest.
  a. Prepare the adequate volume of warmed hepatocyte culture medium containing the compound of interest.
    ***Note:*** 2 mL of solution are enough for triplicates of each experimental condition (enough for 3 × 500 μL replicates for wells with 500 μL of culture medium). Prepare the vehicle controls, also in triplicate.
    **CRITICAL:** before any experiment, make sure you are using non-toxic concentrations to avoid a decrease in cell viability.
  b. Remove the culture media from each well by aspirating it carefully. Avoid touching the bottom of the plate. Since this is the first culture media, discard it.
  c. Add 500 μL of the solution prepared in step 37a to each well.
    ***Note:*** it is important to reduce the time that hepatocytes are without culture medium. Therefore, upon removing the medium from one well, this should be immediately covered with fresh warm medium.
  d. Incubate for a 24-h period.
    ***Note:*** longer incubation periods may be used but the culture should be closely monitored in order to verify that the bottom of the well is covered with cells. Dead hepatocytes detach from the surface, and this can be easily observed under the microscope.
    **CRITICAL:** longer incubation periods will reduce the medium pH and cause cells to detach. The toxicity of experimental drugs will affect the viability of the cells.

38. Upon 24 h incubation, remove the culture medium and replace with new culture medium. See troubleshooting 6.
  a. Prepare the adequate volume of warmed hepatocyte culture medium containing the compound of interest.
  b. Collect the culture media from each well to a pre-labeled 1.5 mL centrifuge tubes. **Do not discard** the culture medium.
    ***Note:*** aspirate the culture medium it carefully and avoid touching the bottom of the plate.
  c. Add 500 μL of the solution prepared in step 38a to each well.
  d. Incubate for 24 h.
  e. Centrifuge the 1.5 mL centrifuge tube of step 38 b at 16,000 × *g* for 10 min at 4°C.
    ***Note:*** each of the collected fraction contains the hepatocyte-derived metabolites.
  f. Transfer the supernatant to clean labeled tubes.
  g. Collect 200 μL and quench with 100 μL of cold acetonitrile, vortex for 5 s, and centrifuged at 16,000 × *g* for 10 min at 4°C to pellet the proteins out of solution.
  h. Analyze the supernatant by UPLC-MS/MS or store at −20°C until analysis. See troubleshooting 1 and troubleshooting 2.
39. Upon 24 h, collect the culture media and replace by new one. Repeat step 38.

***Note:*** culture medium can be replaced as long as the hepatocytes still viable.

Hepatocytes are considered the “gold standard” for *in vitro* metabolism assays due to the closest approximation to hepatocytes *in vivo.*^23^ Despite that, S9 fractions are easier to handle, cheaper, and also contain the major part of both phase I and phase II drug-metabolizing enzymes, whereby this subcellular fraction is a good alternative to metabolic assays.

### Identification of *in vitro* metabolites from cell cultures

**Timing: 0.5–2 h**

In addition to subcellular fractions and hepatocytes, the metabolism in other cells can also be studied. For that, cells are incubated with the drug of interest and metabolites are then collected and analyzed.

**CRITICAL:** before any experiment, make sure you are using non-toxic concentrations to avoid a decrease in cell viability.

Drug metabolites can be extracted using two protocols, according to the adherence characteristics of the cells. Since this protocol intends to identify drug metabolites, no normalization with the control sample is required. Upon extraction, samples were analyzed by UPLC-MS/MS.

#### Adherent cells

**Timing: 0.5–1 h**

This section describes the culture and treatment of adherent cells, followed by cell lysis and metabolite collection for further analysis by UPLC-MS/MS.

***Note:*** this procedure was optimised for a 6-well plate with 1.9 million cells/well. In case of other culture vessels and cell number, solution volumes may need to be adjusted.

40. Expose the cell culture to the drug of interest.
  a. Prepare culture medium containing the drug of interest.
  b. Replace the culture medium of each well with the culture medium prepared in step 40a.
  c. Incubate for desired period of exposure.

***Note:*** prepare vehicle controls in accordance.

41. Wash the cells with 0.9% NaCl solution.
  a. Using a 1 mL pipette or a liquid suction vacuum system, remove the culture medium from each well.
    ***Note:*** culture medium can be processed as described in step 38 (e–h).
  b. Add NaCl solution to each well (e.g., 500 μL to each well of a 6-well plate).
  c. Using a 1 mL pipette or a liquid suction vacuum system, remove the NaCl solution.
42. Add 200 μL of cold methanol:water (4:1 v/v) to each well of a 6-well plate.
43. Place the culture vessel on ice. Scrape the cells into the cold methanol:water solution to aid lysis and their removal, making sure > 90% of the seeded cells are transferred into a 1.5 mL microtube.
44. Wash each well with an additional 200 μL of water (LC/MS grade) and transfer it to the microtube.
45. Centrifuge the microtube at 16,000 × *g* for 30 min at 4°C.
46. Collect the supernatant and analyze by UPLC-MS/MS or store at −20°C until analysis. See troubleshooting 1 and troubleshooting 2.

***Note:*** since we intend to study drug metabolites instead of endogenous metabolites, cells can also be detached with trypsin and metabolites extracted with the following steps (47–55). See In suspension or detached cells.

#### In suspension or detached cells

**Timing: 0.5–1 h**

Cultures in suspension (or detached cells) can also be used in drug metabolism testing. This section describes in detail the required steps to perform the assay.

If cells are in suspension or have been previously detached with trypsin, a freeze/thaw cycle protocol can be employed.

47. Expose the cell culture to the drug of interest.
  a. Prepare culture medium containing the drug of interest.
  b. Replace the culture medium of each well with the culture medium prepared in step 40a.
  c. Incubate for desired period of exposure.

***Note:*** prepare vehicle controls in accordance.

48. Pellet the cells by centrifugation at 150 × *g*, for 10 min at 4°C. Discard the supernatant.
49. Wash the cell pellet with 250–500 μL of 0.9% NaCl solution.
  a. Add 250–500 μL of 0.9% NaCl solution to the cells.
  b. Centrifuge at 150 × *g*, 10 min at 4°C.
  c. Discard the supernatant.
50. Add 500 μL of a cold methanol:acetonitrile:water (2:2:1), and thoroughly mix on a vortex mixer for 20 s.
51. Transfer the microtube to liquid nitrogen for 3 min.
52. Thaw the microtube on ice.
53. Repeat the freeze/thaw cycle at least 2 more times.
54. Centrifuge be at 16,000 × *g* for 15 min at 4°C and collect the supernatant for analysis.
55. Collect the supernatant and analyze by UPLC-MS/MS or store at −20°C until analysis. See troubleshooting 1 and troubleshooting 2.

## Expected outcomes

The present protocol describes the production and identification of montelukast metabolites using isolated enzymes and various *in vitro* systems: recombinant human cytochrome P450 systems (CYP3A4, 2C8, and 2D6) expressed in baculosomes together with NADPH:P450 oxidoreductase, hydrogen peroxide-dependent peroxidases and tyrosinase, and subcellular fractions (microsomal, cytosolic, and S9 fraction) from different tissues. Primary hepatocytes have also been used to obtain a cellular model for drug metabolism. All the products obtained and their identification are described in detailed in Marques et al.^1^

Herein, we present a short example of some metabolites (produced and expected) according to phase I and phase II metabolism.

**Figure 2:**
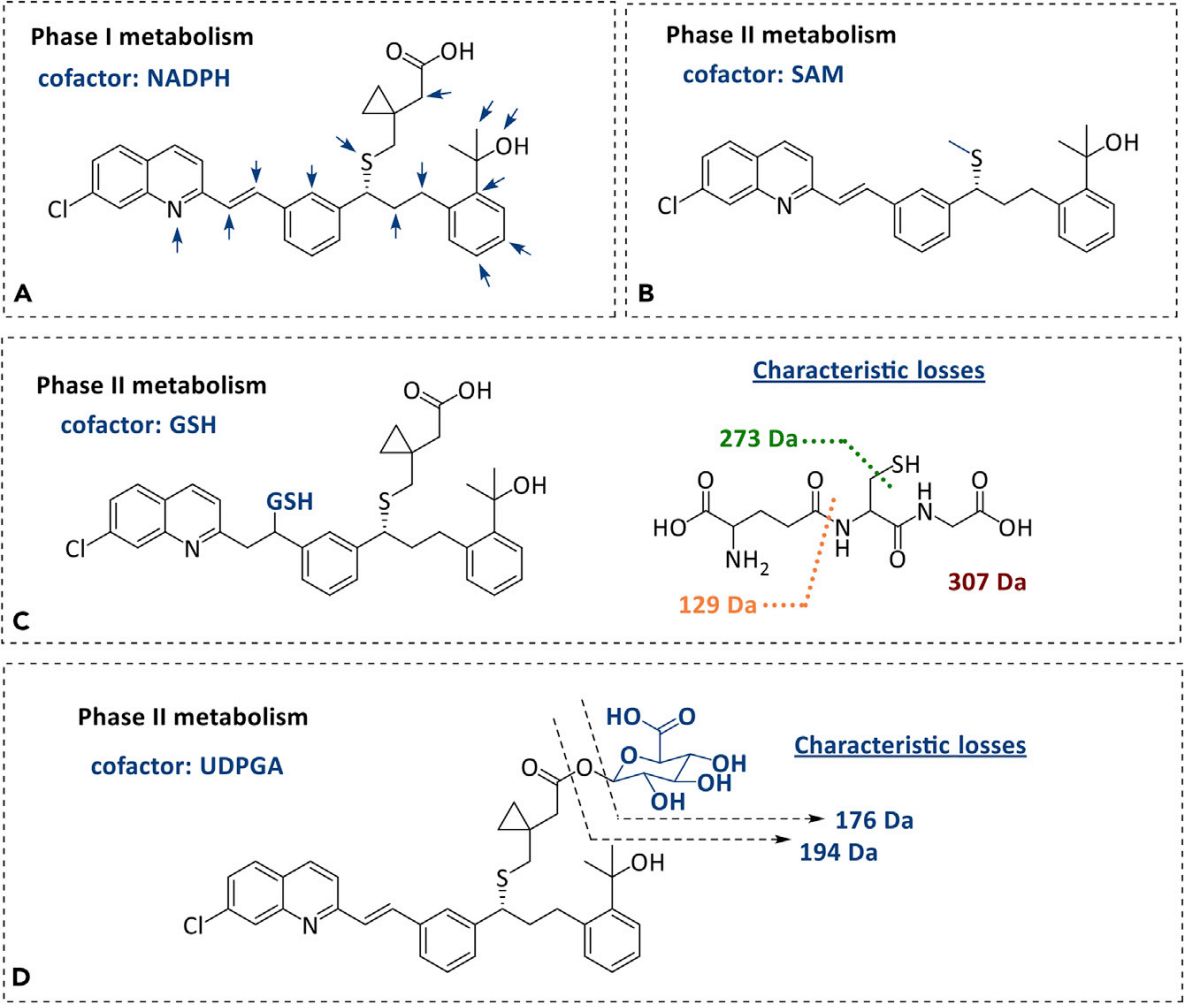
MTK metabolites identified using the systems described This figure summarizes some of the MTK metabolites identified using the systems described in this paper, showing some characteristic losses of these metabolites. (A) Phase I metabolites: the arrows show the different positions where oxidation reactions have been identified as having been modified. (B) Phase II metabolite upon methylation. (C) Glutathione conjugation with montelukast. Characteristic losses of the GSH moiety are presented on the right side of the panel. (D) Glucuronidation product of montelukast, as well as the characteristic losses of the glucuronide.

Phase I metabolites consist mainly in products of oxidation, epoxidation, carbon hydroxylation, *N*-hydroxylation, and *N*-oxidation, and are characterized by an increase of +16 Da. Demethylation products can also be obtained and correspond to a decrease of -14 Da, as well as oxidative desulfuration derivatives (-32 Da) and phosphorous oxidation derivatives (-14 Da). Phase I metabolites can also include hydrolytic and reduction products. Figure 2 illustrates some of these metabolites.

While phase I metabolism is focused on oxidation and reduction reactions in order to convert lipophilic drugs into more polar molecules, phase II metabolism intends to further increase the polarity of compounds, facilitating its excretion. For that, phase I metabolites (or the drug itself) is conjugated with other groups (e.g., UDPGA (+176 Da), PAPS (+80 Da), GSH (+307 Da), A-CoA (+42 Da), SAM (+14 Da), and glycine (+57 Da)) in the presence of different enzymes (e.g., UDP-glucuronosyltransferase, sulfotransferase, glutathione *S*-transferase, acetyl-transferase, methyl transferase, and glycine *N*-acyltransferase, respectively).

Figure 2 illustrates some of these phase II metabolites, as well as the characteristic losses of glutathione conjugates (307 Da (GSH), 273 Da (GSH-S), or 129 Da (γ-glutamyl) Da) and glucuronides (176 Da and 194 Da).

**Figure 3:**
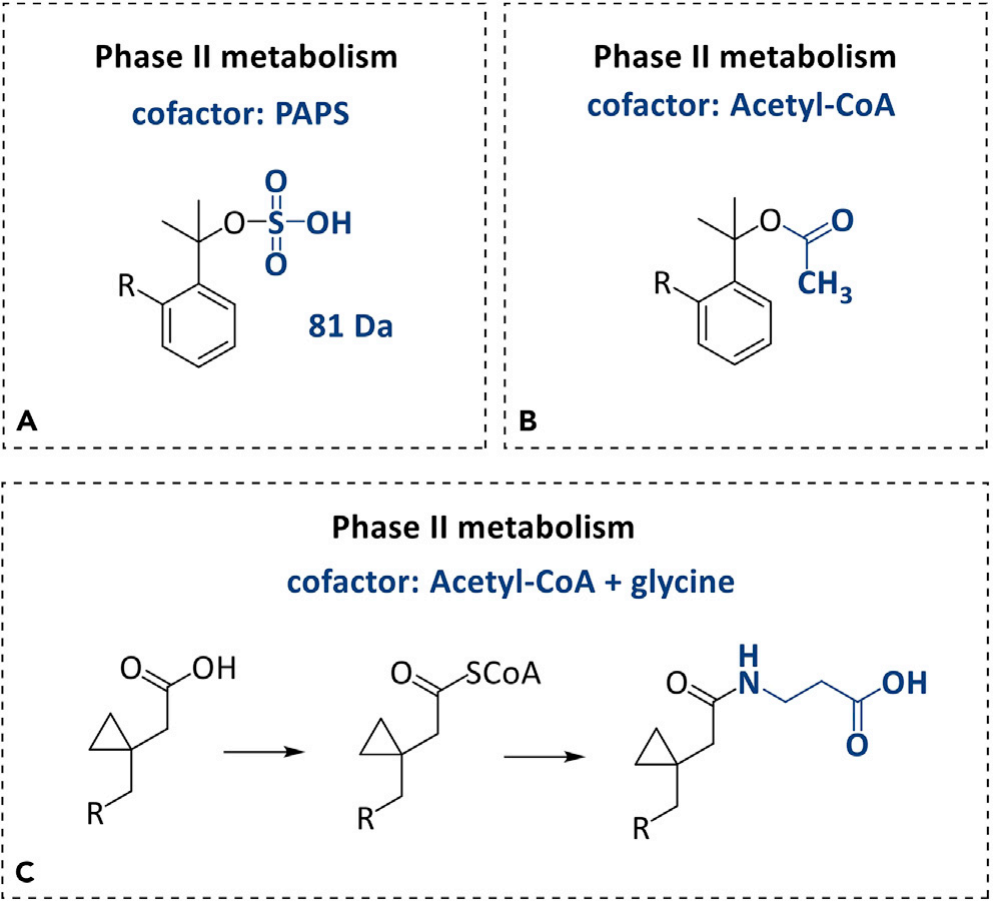
Possible phase II metabolites This figure illustrates plausible metabolites that could be formed during phase II metabolism. None of these metabolites were found in MTK incubations. (A) Expected metabolite produced by sulfate conjugation. (B) The acetylation of drugs can occur in the NH_2_ or OH moieties and is a two-step reaction, similar to the represented in (C). (C) The amino acid conjugation product with glycine.

Figure 3 exemplifies other plausible phase II metabolites such as sulfonylation products and glycine conjugation. However, they were not found with montelukast.

## Quantification and statistical analysis

Metabolite identification was performed employing a mass spectrometry-based approach. Reaction products were analyzed using an Elute UHPLC system coupled to an Impact II QqTOF mass spectrometer with an electrospray ion source. Metabolites were separated on a reverse-phase column Luna 3 μm C18(2) (100 Å, 150 × 2 mm) at a constant temperature of 35°C, using a gradient elution at a flow rate of 200 μL/min (mobile phases A: 0.1% (v/v) formic acid in water (ESI+) or 5 mM ammonium acetate pH 6.5 (ESI-); mobile phase B: 0.1% (v/v) formic acid in acetonitrile): 0–4 min, 5% B; 4–22 min, 5%–100% B; 24 min, 100% B; 24 25 min, 100 to 5% B, followed by a 10 min column re-equilibration step.

High resolution mass spectra were acquired in both ionization modes, with the following acquisition parameters: capillary voltage, 4.5 kV (ESI+) or 3 kV (ESI-); end plate offset, 500 V; nebulizer, 2.0 bar; dry gas (N2) flow, 8.0 L/min; dry heater temperature, 220°C. The tune parameters were set according to: transfer funnel 1/2 RF power (150/200 Vpp), hexapole RF power (50 Vpp), ion energy (4.0 eV), low mass (50 m/z), collision energy (7.0 eV), collision RF power (650 Vpp), transfer time (80 μs), pre-pulse storage (5 μs). Spectra acquisition were performed with an absolute threshold of 25 counts per 1,000. MS/MS spectra with an *m/z* scan range from 50 to 1,500 were acquired with a 2.00 Hz spectra rate. Thresholds for auto MS/MS were set at 400 counts per 1,000, with a limit of 3 precursors and exclusion after 3 spectra. All acquisitions were performed with an *m/z* range from 50 to 1,500 and with a 2 Hz spectra rate.

Raw data were converted to mzXML format with ProteoWizard MSConvert,^18^ and the search for MTK i*n vivo* metabolite was performed using MZmine v3^16^^,^^17^ with an in-house library of MTK metabolites based on the predicted metabolites. Full parameters for MZmine search are provided below for the latest version of MZmine (v3). These values have been derived in our group for the Bruker ImpactII QqTOF throughout time; a full explanation of the MZmine algorithm and parameters can be found online (https://mzmine.github.io/mzmine_documentation/workflows/lcmsworkflow/lcms-workflow.html).

1. Mass detection. This step scans the data files and generates a list of *m/z* values (mass list) in each scan in each sample.
  a. Mass detection parameters: mass detector, centroid; noise level, 1,000 (positive ionization mode), 50 (negative ionization mode).

***Note:*** the noise level is highly dependent on the experimental conditions, the purity of the eluents used, and the background of the samples. These values must be chosen so that the baseline of the chromatograms is below the noise threshold, but all chromatographic peaks are above that level.

2. Chromatogram building. For each *m/z* value, scans are processed to obtain an extracted ion chromatograms (EICs) for each mass value. The most widely used method is the *ADAP chromatogram builder.*
  a. ADAP chromatogram builder parameters: minimal group size in number of scans, 2; group intensity threshold, 50; minimal highest intensity, 50; scan to scan accuracy (*m/z* tolerance), 3 mDa.

***Note:*** the minimal group size parameter depends on the sampling frequency (from the data acquisition parameters for the mass spectrometer) and on the peak duration. For example, at a 3 Hz frequency, a peak with a 0.1 min duration (6 s) as least 18 spectra. Using an higher value gives a lower total count of extracted chromatograms (EICs).

***Note:*** an *m/z* tolerance of 0.003–0.005 (3–5 mDa) is adequate for most TOF experiments. A higher tolerance will lead to more identified compounds but at the cost of possible misidentifications. It is recommended that the tolerance is set as *m/z* and not ppm; setting either value to 0 means it will be disregarded.

3. Chromatogram smoothing. This is an optional step, recommended to improve the shape of the peaks present in the various EICs, which leads to a simpler downstream processing.
  a. Smoothing parameters: algorithm, Savitzky Golay; retention time smoothing, 5.
4. Local minimum feature resolver. This module aims to resolve co-eluting and overlapping peaks. While most parameters presented here work across a large set of experiments, in some cases it is necessary to adjust the minimum search range RT to improve the resolution of overlapping peaks. Full details for these parameters are given online (https://mzmine.github.io/mzmine_documentation/module_docs/featdet_resolver_local_minimum/local-minimum-resolver.html).
  a. Parameters: retention time tolerance, 0.15 min; MS1 to MS2 precursor tolerance, 3 mDa; limit by RT edges, yes; dimension, retention time; chromatographic threshold, 90.0%; minimum search range rt, 0.05 min; minimum relative height, 0.0%; minimum absolute height, 1,000; minimum ratio of peak top/edge, 1.80; minimum number of data points, 4.
5. Isotope filtering. Isotope resolving with the ^13^C isotope filter (isotope grouper) allows eliminating redundant data coming from ^13^C isotopologues, giving a more sensitive detection of possible isotope signals from other elements. In alternative, a search for isotopic signals from other elements can be performed using the isotope finder module. While both can be used sequentially, the isotope finder module has priority over the isotope grouper model.
  a. ^13^C isotope filter (isotope grouper) parameters: *m/z* tolerance, 5 mDa; retention time tolerance, 0.05 min; monotonic shape, yes; maximum charge, 1; representative isotope, lowest *m/z.* If ions with charger greater than 1 are expected, given the drug’s nature and possible metabolic pathways, increase the maximum charge parameter adequately.
  b. Isotope peak finder parameters: Chemical elements, setup as required for the tested drugs and the expected metabolites; *m/z* tolerance, 1.5 mDa; maximum charge of isotope *m/z*, 1.
6. Join aligner. This module will align detected peaks in different samples.
  a. Parameters: *m/z* tolerance, 5 mDa; retention time tolerance, 0.1 min; weight for *m/z*, 3; weight for retention time, 1.

***Note:*** If peak drifting between samples becomes apparent, retention time tolerance must be improved. We obtained better identification results with a 0.2 min tolerance.

***Note:*** The relative weights of *m/z* and rt must account for the experimental conditions and results. While 3 and 1 values are indicated as default for UHPLC-TOF experiments, no changes were observed using 1 and 0 values.

7. Annotation / custom database search. Following these data processing, peak identification was performed resorting to an in-house built database. Other processing options are possible, particularly the application of various filters, for which documentation is available online (https://mzmine.github.io/mzmine_documentation/workflows/lcmsworkflow/lcms-workflow.html). This database can be created in any spreadsheet and saved as a csv file. A sample table is given below.
  a. Parameters for custom database search: m/z tolerance, 10 mDa; retention time tolerance, 0.0 if you have no values for standard of expected metabolites. You must indicate which information you want to import from the database file and match each column name in the csv file (first row) to the MZmine data types. Required values are Neutral mass (monoisotopic mass for the parent compound), Precursor *m/z* (monoisotopic mass for the protonated or deprotonated precursor), Formula (chemical formula of the parent compound), and Compound, which match to the columns below titled “neutral”, “precursor”, “formula”, “name”.

**Table:** 

Neutral	Precursor	Formula	name
78.046402	79.054227	C_6_H_6_	Benzene
94.041316	95.049141	C_6_H_6_O	Phenol
  b. Monoisotopic masses can be computed with various software packages for any given formula. Online calculators are also available (for example, this one (https://bmrb.io/metabolomics/mol_mass.php?formula=&subaction=Specified+Composition&updateIsoComp=1&1H=99.9885&2H=0.0115&3H=0&16O=99.757&17O=0.038&18O=0.205)).

## Limitations

The major limitation to this protocol is the potential protein binding of the tested drugs. If the drug has a high protein binding, no protein quenching or centrifugation should be performed, because most drug metabolite will be protein-associated and be precipitated out of the solution. In those cases, samples should be analyzed directly from the reaction mixture.

**CRITICAL:** the analysis of reaction mixtures without protein precipitation may reduce column lifetime. The use of a guard column is highly recommended in order to prolong the life of the chromatographic column.

## Troubleshooting

### Problem 1

Absence or low abundance of drug metabolites in reaction mixtures.

After incubation with enzymes, drug metabolites are not found or are present in low abundance (steps 7, 15, 23, 38h, 46, and 55 of the step-by-step method details).

### Potential solution

Some of the possible causes for this problem include an inadequate incubation time, metabolite protein binding, or low enzymatic activity. However, it is important to consider that the expected metabolites may not be produced in the experimental conditions tested.

In the case of *in vitro* metabolites produced in cell culture, a low number of cells could also be associated with a low abundance of metabolites.

### Problem 2

Poor chromatographic separation.

During the liquid chromatography step, the chromatographic separation does not allow a correct separation of the metabolites present in the reaction mixture (steps 8, 16, 24, 38h, 46, and 55).

### Potential solution

This problem can be caused by the incompatibilities between mobile phases and chromatographic column and the polarity of metabolites.

Make sure the selected chromatographic conditions are the most adequate for the parent drug.

### Problem 3

Low cell viability.

After thawing, the yield of viable cells is low (step 31).

### Potential solution

The low viability of cells can be due to an improper cell handling (e.g., more than 2 min at 37°C, or a rough handling of the hepatocytes during the hepatocytes dispersion in culture medium).

Make sure the hepatocytes are handled with care. Moreover, ensure that the cell suspension is homogenous before counting the cells.

### Problem 4

Cells fail to attach.

After plating, cells take too long to adhere to the tissue culture plate (step 35).

### Potential solution

This problem can occur due to an incorrect washing step during the collagen coating (collagen coating of 24-well tissue culture plates). Make sure the hepatocytes are plateable.

### Problem 5

Cells do not reach optimal monolayer confluency.

After plating, cells do not reach confluency (step 36).

### Potential solution

A low number of viable cells with consequent low cell density could be the major reason for the sub-optimal monolayer confluency. However, a low attachment and an insufficient dispersion of hepacotcytes (step 35) can also be involved.

If cell density is too high, cell clumps will be formed, as well as an increase of debris in solution.

### Problem 6

The abundance of metabolites produced by hepatocytes is decreasing.

After 24 h of exposure to drugs, the abundance of metabolites produced by hepatocytes is decreasing (step 38).

### Potential solution

The decrease of produced metabolites could be caused by a decrease in cell viability or a decrease in cell number. Some of the reasons involved include the reduction of the medium pH, cell detachment, or toxicity induced by the drug in test or a produced metabolite. Enzyme inhibition can also decrease the rate of drug metabolism.

## Resource availability

### Lead contact

Further information and requests for resources and reagents should be directed to and will be fulfilled by the lead contact, Cátia Marques (catiafmarques@tecnico.ulisboa.pt).

### Materials availability

This study did not generate new unique reagents.

## Data Availability

Data generated using the procedures presented in this paper are published in Marques et al.^1^
